# Extrachromosomal DNA Amplification as a Prognostic Factor for Cancer

**DOI:** 10.3390/jpm16060316

**Published:** 2026-06-12

**Authors:** Filip Gajewski, Joanna Pec, Jakub Kleinrok, Weronika Pająk, Katarzyna Pacyna, Agata Tokarzewska, Paweł Krawczyk

**Affiliations:** 1 Student Scientific Association, The Department of Pneumonology, Oncology and Allergology, Medical University of Lublin, Doktora Kazimierza Jaczewskiego 8, 20-090 Lublin, Poland; 2 Chair and Department of Clinical Pathomorphology, Medical University of Lublin, Kazimierza Jaczewskiego 8b, 20-090 Lublin, Poland; 3 Department of Pneumonology, Oncology and Allergology, Medical University of Lublin, Doktora Kazimierza Jaczewskiego 8, 20-090 Lublin, Poland

**Keywords:** extrachromosomal DNA (ecDNA), oncogene amplification, tumour heterogeneity, cancer prognosis, drug resistance, genomic instability, biomarker, targeted therapy

## Abstract

Background: Extrachromosomal DNA (ecDNA) amplification represents a distinct mechanism of genomic instability in cancer, increasingly recognized for its role in aggressive disease progression. This review examines how ecDNA drives tumour evolution and assesses its potential as both a prognostic marker and therapeutic target. Methods: The authors integrate findings from multiple detection platforms—including FISH, whole-genome sequencing, and specialized reconstruction algorithms—and present data across diverse cancer types; no preregistration is noted, and no animal studies are included. Results: ecDNA consists of circular, acentric DNA elements carrying high-copy oncogene amplifications (such as *EGFR*, *MYC*, *MDM2*, and *CDK4*). Unlike chromosomal DNA, ecDNA segregates unevenly during cell division, generating intratumoral heterogeneity, accelerating adaptation to selective pressures, and promoting resistance to therapy. Pan-cancer surveys summarized here reveal ecDNA in a significant subset of tumours, with particularly high frequencies in liposarcoma, glioblastoma, and HER2-positive breast cancer, and consistent associations with worse clinical outcomes. Conclusions: The authors conclude that ecDNA amplification serves as a credible adverse prognostic indicator and holds promise for refining risk stratification and guiding treatment strategies. However, they stress that clinical adoption remains constrained by the absence of standardized, scalable, and reproducible detection.

## 1. Introduction

### 1.1. Background on Cancer and Genomic Instability

The basis of cancer is the accumulation of genetic and epigenetic changes that lead to disorders in the control of proliferation, apoptosis and cell survival. A fundamental feature of cancer, responsible for the accumulation of genetic changes, is genomic instability. This phenomenon promotes the development of tumour cell heterogeneity, which allows for the selection of clones with a growth advantage, greater aggressiveness and the ability to evade therapeutic mechanisms [1,2,3,4]. The source of genomic instability is the increased frequency of point mutations, chromosomal rearrangements and changes in gene copy number. These alterations may occur not only in chromosomal DNA but also in extrachromosomal DNA (ecDNA) [5,6,7]. Cancer remains one of the leading causes of morbidity and mortality worldwide and represents a major global public health challenge. According to the most recent GLOBOCAN 2022 estimates, there were nearly 20 million new cancer cases and approximately 9.7 million cancer-related deaths worldwide in 2022. Lung cancer was the most frequently diagnosed cancer and the leading cause of cancer mortality globally, followed by female breast, colorectal, prostate and stomach cancers among the most commonly diagnosed malignancies. Demographic projections further indicate that the number of new cancer cases may increase to approximately 35 million by 2050, mainly as a consequence of population growth and ageing. These data highlight the increasing clinical and socioeconomic burden of cancer and emphasize the need for a better understanding of the molecular mechanisms that drive tumour initiation, progression and therapeutic resistance [8].

### 1.2. History of ecDNA

**Figure 1:**
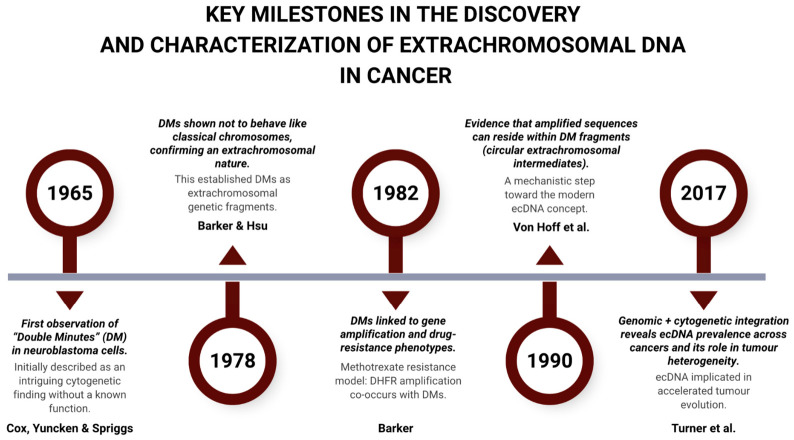
Key milestones in the discovery and characterization of extrachromosomal DNA (ecDNA) in cancer. The timeline summarizes major historical steps in the understanding of ecDNA, from the first cytogenetic observations of double minutes in neuroblastoma cells, through the association of double minutes with gene amplification and therapy resistance, to genomic studies demonstrating the recurrent presence of ecDNA across multiple cancer types and its role in tumour heterogeneity and accelerated evolution [9,10,11,12,17].

The first reports on ecDNA date back to the 1960s. The starting point is considered to be a paper published by David Cox et al., which described the phenomenon for the first time. The phenomenon was only an observation and was not linked to any function at that time [9]. In the 1970s, it was discovered that DMs do not behave like classical chromosomes, confirming that they are extrachromosomal genetic fragments [10]. In the 1980s, DMs were linked to gene amplification and therapy-resistant phenotypes. The breakthrough came when it was shown that DMs can carry amplified genes in a model of methotrexate resistance, where amplification of the *DHFR* gene co-occurred with DMs [11]. An important milestone was the work by Von Hoff et al. (1990), which provided evidence that amplified sequences can occur in DM fragments [12]. However, the real breakthrough was the work of Turner et al. (Nature, 2017), which combined genomic and cytogenetic data, indicating the frequency of ecDNA in many types of cancer and its role in tumour heterogeneity and accelerated evolution [13] (Figure 1).

Although ecDNA is most often discussed in the context of cancer, extrachromosomal circular DNA (eccDNA), a broader category of circular DNA molecules, is not exclusively a pathological phenomenon. Under normal physiological conditions, smaller extrachromosomal circular DNA molecules, referred to as eccDNA, represent a broader category that should be distinguished from large oncogene-bearing ecDNA, have been detected in different eukaryotic cells and tissues. These molecules differ from large oncogene-bearing cancer-associated ecDNA molecules, because they are usually smaller, more heterogeneous and do not necessarily carry amplified oncogenes. Their presence suggests that circular extrachromosomal DNA may represent a natural component of genome biology rather than only a marker of malignant transformation. Recent studies indicate that eccDNA may participate in several physiological processes. Its proposed functions include regulation of gene expression, production of regulatory RNAs, modulation of genome plasticity and involvement in cellular responses to stress. eccDNA may also contribute to innate immune signalling, for example, through activation of DNA-sensing pathways, and has been linked to ageing-related biological processes. However, the physiological role of eccDNA remains incompletely understood and should be clearly distinguished from the pathological accumulation of large ecDNA molecules carrying oncogenes in cancer cells [14,15,16].

### 1.3. Purpose and Scope of Work

Currently, we are seeing an increase in reports indicating that the presence of ecDNA correlates with poor prognosis, shorter overall survival and increased therapy resistance. This paper aims to discuss how ecDNA affects tumour progression, whether it can be used as an independent prognostic marker, and what its potential clinical significance is in future cancer diagnostics and treatment.

## 2. Biological Characteristics of ecDNA

### 2.1. Structure and Defining Features of ecDNA

ecDNA is a circular, double-stranded DNA. It exists independently of the canonical chromosomal complement within the cell nucleus. ecDNA molecules are typically large and lack centromeres or telomeres. These features distinguish ecDNA from linear chromosomal DNA and contribute to its non-canonical behaviour in tumour cells. ecDNA often contains full-length oncogenes and their associated regulatory sequences, which gives it increased transcriptional potential compared to equivalent genomic loci due to increased chromatin accessibility. This enhances accessibility and contributes to the hyperactive oncogene expression that drives tumour progression and malignant phenotypes [17,18].

A study by Kim et al. highlights that ecDNA is commonly found in different types of cancers. However, it rarely occurs in healthy tissues, which emphasizes its association with cancer transformation and disease progression [19].

The circular structure of ecDNA may support independent replication and facilitate the reorganization of regulatory elements. It helps ecDNA expand the transcriptional repertoire of cancer cells beyond the limitations of chromosomal architecture [18].

### 2.2. Mechanisms of ecDNA Formation

The formation of ecDNA is a multistep process, directly linked to genomic instability and DNA rearrangements. It involves several distinct but partially overlapping mechanisms. Circular DNA fragments, which later turn into ecDNA, are generated during multiple processes.

(1) Chromothripsis

Chromothripsis is one of the best-characterized mechanisms of ecDNA formation. It involves a single catastrophic event in which chromosomes undergo extensive fragmentation followed by random rejoining of chromosomal fragments. During this process, some DNA fragments are not integrated into chromosomes but instead circularize to form ecDNA. These circular fragments frequently retain oncogenes and regulatory elements, which contribute to their functional significance in cancer progression [17,20].

(2) Breakage–Fusion–Bridge (BFB) Cycles

Another mechanism responsible for ecDNA formation is the BFB cycles. They are initiated by telomere shortening or dysfunction, leading to end-to-end chromosome fusions and the formation of dicentric chromosomes. During mitosis, mechanical stress causes these chromosomes to break unevenly, generating amplified DNA segments that may subsequently be excised and circularized into ecDNA. Repeated BFB cycles further increase genomic rearrangements and amplification levels [14,21].

(3) Replication-Based Mechanisms

Replication-based mechanisms also play a crucial role in ecDNA formation. Replication fork stalling, template switching, and abnormal repair of double-strand breaks can lead to complex genomic rearrangements. These processes generate ecDNA fragments through aberrant replication and misalignment of homologous sequences, facilitating their cyclization of chromosome ends [21,22].

(4) Excision of Oncogenic Regions

Importantly, ecDNA formation may also occur via the excision of chromosomal regions containing super-enhancers and oncogenes, followed by their circularization. This process enables the creation of highly transcriptionally active ecDNA hubs, which can dynamically regulate oncogene expression and contribute to tumour heterogeneity [23].

**Figure 2:**
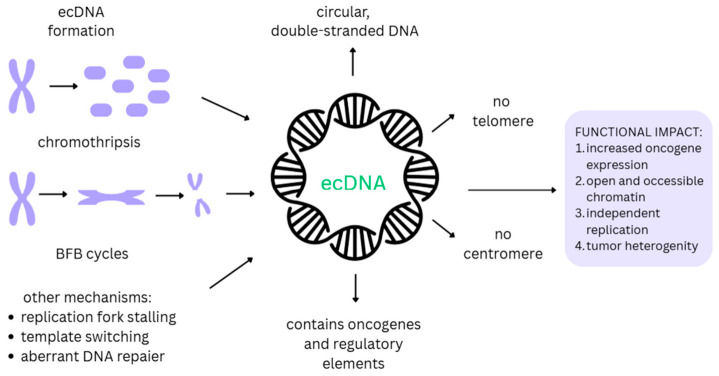
A summary of ecDNA formation and main characteristics.

Collectively, these mechanisms emphasize that ecDNA formation is not the result of a single pathway but rather an interaction between DNA damage responses, replication stress, and structural genome rearrangements in the formation of ecDNA in cancer cells. The main mechanisms of ecDNA formation, together with its defining features and functional consequences, are summarized in Figure 2.

The scheme summarizes selected mechanisms that may generate ecDNA, including chromothripsis, break-fusion-bridge cycles, replication fork stalling, template switching, and aberrant DNA repair. The central circular structure represents ecDNA, which typically lacks centromeres and telomeres, may contain oncogenes and regulatory elements, and can replicate independently. These structural properties contribute to increased oncogene expression, open chromatin organization, non-Mendelian inheritance, and intratumour heterogeneity.

### 2.3. Inheritance Dynamics and Tumour Heterogeneity

A characteristic feature of ecDNA is its non-Mendelian inheritance. Unlike chromosomal DNA, ecDNA does not have centromeres and does not attach to the mitotic spindle in a controlled manner. It results in ecDNA molecules being unevenly segregated during mitosis, and later uneven distribution of copy numbers among daughter cells [24].

This non-Mendelian inheritance promotes genetic diversity within tumours, as different cell subclones can accumulate different levels of ecDNA-mediated oncogene amplification. When tumours face selective pressures, such as cancer therapy, cells that possess more advantageous oncogene copy levels are more likely to survive, driving tumour progression and the emergence of treatment resistance [25].

**Figure 3:**
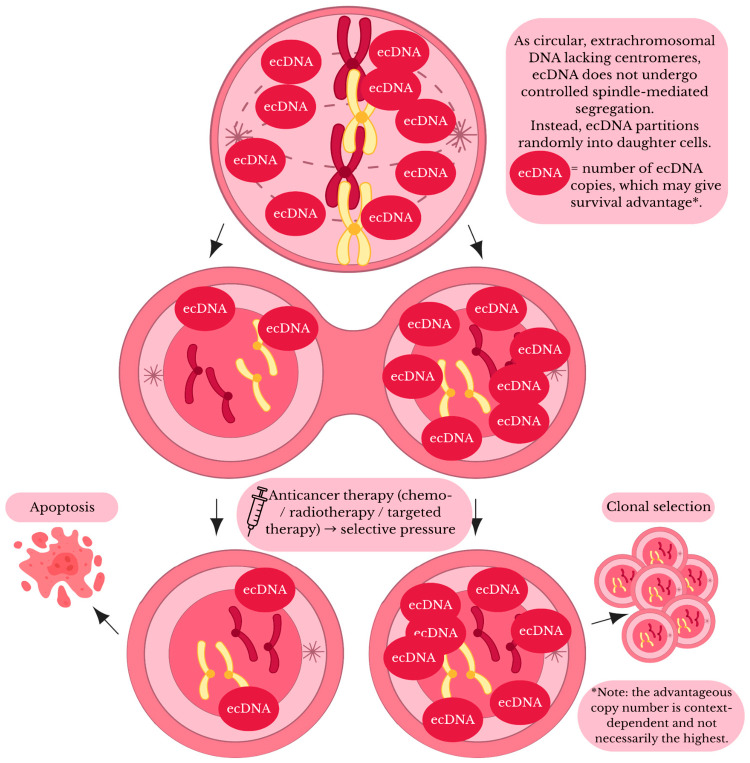
Random ecDNA segregation drives intratumour heterogeneity and therapy selection. The figure illustrates how acentric ecDNA molecules carrying amplified oncogenes are unevenly distributed during mitosis because they do not segregate through the canonical centromere-dependent mechanism. As a result, daughter cells may inherit different ecDNA copy numbers, leading to variable oncogene dosage and transcriptional output. Under therapeutic pressure, subclones with ecDNA configurations that provide a survival advantage may be preferentially selected, whereas more sensitive cells may undergo apoptosis. This process contributes to intratumour heterogeneity, tumour evolution, and the emergence of treatment-resistant clones. Red circular elements represent ecDNA molecules; chromosomes are shown as linear intranuclear structures; arrows indicate the direction of mitotic segregation and subsequent clonal selection.

Recent studies also suggest that multiple types of ecDNA within a tumour cell may undergo coordinated co-segregation, allowing different oncogenes and enhancers to be amplified at the same time [26]. This phenomenon further enhances phenotypic heterogeneity and may facilitate the emergence of novel gene-regulatory architectures that are maintained across multiple rounds of cell division (Figure 3).

### 2.4. Differences Between Gene Amplification on ecDNA and Chromosomal Amplification

Gene amplification via ecDNA differs from chromosomal amplification in both inheritance patterns and functional consequences. Chromosomal amplifications, such as homogeneously staining regions (HSRs), follow canonical mitotic segregation, resulting in relatively stable distribution across daughter cells. In contrast, ecDNA lacks centromeres and segregates unevenly during cell division, leading to increased intratumoral heterogeneity [13].

ecDNA amplification is also characterized by dynamic changes in gene copy number. ecDNA molecules can rapidly increase or decrease in number, enabling flexible regulation of oncogene dosage. This contrasts with chromosomal amplification, which is generally more stable.

Functionally, ecDNA-driven amplification is associated with higher levels of oncogene expression compared to chromosomal amplification. This is partly due to increased chromatin accessibility and the ability of ecDNA to reorganize regulatory elements, enhancing transcriptional activity [19].

Oncogenes located on ecDNA show increased impact on transcription compared to the same genes not associated with ecDNA. This high transcriptional activity is promoted by greater chromatin accessibility in ecDNA, as well as increased contact with active ultra-long-range chromatin [23].

The increased expression of oncogenes is also associated with ecDNA, which is influenced by the formation of clusters of ecDNA, called hubs, in the nucleus. These hubs consist of approximately 10–100 ecDNAs within the cell nucleus. Hubs enable intermolecular interactions between genes and transcription enhancers [27].

In the work of Zhu et al., the authors also suggested that ecDNA may act as a mobile transcription enhancer, thereby promoting tumour progression. ecDNAs can transfer regulatory elements (i.e., enhancers) to the vicinity of chromosomal genes, causing a global increase in transcription, including that of oncogenes [28].

Together, these mechanisms suggest that ecDNA may contribute to a more aggressive tumour phenotype by increasing oncogene transcription [23,27] and promoting intratumoural heterogeneity [24].

## 3. ecDNA Detection Methods

Detection of ecDNA usually combines a screening step with a confirmation method, because no single assay is simultaneously high throughput, structurally resolved, and widely available in routine molecular tests [29].

At the cytogenetic level, fluorescence in situ hybridization (FISH) enables direct visualization of extrachromosomal amplification and can distinguish extrachromosomal signals from chromosomal loci with high specificity [13]. However, FISH is less suited for large cohorts due to limited throughput and the need for probe design targeted to specific genomic oncogenes or regions.

Sequencing-based detection is dominated by next-generation sequencing (NGS) coupled with deep bioinformatics analysis. Using whole-genome sequencing (WGS), ecDNA can be inferred from breakpoint patterns and copy-number structure, and tools like AmpliconArchitect are widely used to reconstruct focal amplification architectures consistent with ecDNA [30]. In practice, broad deployment of WGS remains constrained by cost and the computational/analytical burden, especially in retrospective clinical cohorts [30,31].

Circle-seq enriches the broader pool of extrachromosomal circular DNA molecules, which also includes ecDNA. Therefore, it can be used as a screening approach, but its findings should be confirmed with ecDNA-oriented molecular methods [30,32]. For higher-resolution genetic assays, CRISPR-based capture approaches such as CRISPR-CATCH can enrich selected ecDNA molecules for detailed profiling, but require advanced technical workflows [33].

Because RNA-seq is far more accessible than WGS in many clinical datasets, transcriptome-derived surrogates are increasingly attractive for screening of ecDNA. Ju et al. [29] proposed a 12-gene expression-based assay to predict ecDNA status from RNA-seq and confirmed predictions experimentally using FISH in both cell lines and clinical samples, supporting a logical RNA-seq screen and then FISH/WGS confirmation strategy.

The main approaches used for ecDNA detection and characterization are summarized in Table 1, highlighting their practical strengths and limitations.

As ecDNA detection in terms of cancer diagnosis seems to be a very promising tool for diagnostic accuracy improvement, we need to remain aware of the presence of technical limitations. In experimental models, liquid biopsy material is the main medium used for ecDNA detection. There are various techniques involved in ecDNA detection and quantification, including electron microscopy, 2D gel electrophoresis, inverse PCR, fluorescence in situ hybridization [abbrev. FISH], density gradient centralization [34], along with more advanced technologies, such as whole-genome sequencing, single-cell sequencing and circulating DNA capture methods. These technologies allow scientists to obtain more thorough insight into the genetic heterogeneity of ecDNA, making the identification of tumour-specific ecDNA signatures easier. We need to be aware of the limitations borne by each of the methods. As more advanced technologies prove to be more insightful, there are major cost and time prohibitions, including advanced training of diagnostic professionals. More cost-effective methods prove to have lower sensitivity, making the detection of low-abundance ecDNAs more challenging. Therefore, along with more complex research regarding ecDNA itself, there is a need for subsequent research, allowing the development of diagnostic technologies overcoming these shortcomings [34,35].

## 4. Amplification of ecDNA in Tumours

### 4.1. Recurrently Amplified Oncogenes on ecDNA

Genomic studies based on whole-genome sequencing have demonstrated that ecDNA is a significant mechanism for generating high-level focal oncogene amplification across various cancers [13,19].

Kim et al., in pan-cancer whole-genome sequencing analysis of 3212 tumour samples and 1810 normal samples across 29 cancer types, reported that 38% of the 24 most recurrently amplified oncogenes were most frequently present on circular amplicons consistent with ecDNA. This proportion increased to 53.5% for highly amplified oncogenes with a copy number greater than eight. The analyzed recurrently amplified oncogenes included key drivers such as *EGFR*, *MDM2*, *MYC*, *CDK4*, *ERBB2*, *SOX2*, *TERT* and *CCND1*, among others [13].

Functionally, ecDNA amplification is associated with exceptionally strong transcriptional output. Oncogenes carried on ecDNA, such as *EGFR*, *MYC*, *MDM2* and *CDK4*, are among the top 1% of highly expressed genes in cancer transcriptomes [27]. The high ‘potency’ of these ecDNA-associated oncogenes results from their high copy number and the regulatory features of ecDNA [23,27,36].

#### 4.1.1. EGFR Gene

*EGFR* is one of the most frequently amplified oncogenes across multiple cancer types [36]. In their study on glioblastoma multiforme (GBM), de Carvalho et al. emphasized that the *EGFR* amplification plays a significant role in driving this disease. When carried by ecDNA, the functional significance of *EGFR* increases [37]. By activating key pathways, such as the RAS–RAF–MEK–ERK (MAPK) and PI3K–AKT–mTOR pathways, *EGFR* influences the increased proliferation and growth of cancer cells, as well as the inhibition of apoptosis [38]. *EGFR* transmitted by ecDNA may be unevenly distributed among cells. This variability in *EGFR* copy number may contribute to treatment difficulties [13,37].

#### 4.1.2. MYC Gene

*MYC* is a gene that encodes a transcription factor, and its deregulation is associated with one of the key mechanisms of cancer transformation. It acts by selectively increasing the expression of genes that promote growth and proliferation. It also affects metabolic pathways and nutrient uptake, leading to increased tumour mass. In cancer, deregulation of *MYC* and loss of checkpoints result in sustained activity of *MYC*-dependent growth and metabolic programmes [39]. In selected cancers, *MYC* amplifications can be transmitted by ecDNA, which increases the *MYC* dose. In their study, Pongor et al. demonstrated that ecDNA amplifications function as *MYC*-amplifying units, promoting high intratumoural heterogeneity and transcriptional plasticity, and that the presence of complex ecDNA amplifications is associated with poorer clinical outcomes in patients with small cell lung cancer (SCLC) [40].

#### 4.1.3. MDM2 and CDK4 Genes

The *MDM2* oncogene encodes an E3 ubiquitin ligase. It is an important negative regulator of p53, directing it to proteasomal degradation. Overexpression of *MDM2* silences the p53 response [41,42]. In contrast, the *CDK4* oncogene encodes a cyclin-dependent kinase that promotes entry of cells into the S phase and, consequently, their proliferation [30]. *MDM2* and *CDK4* gene amplification are often discussed in the context of adipose tissue tumours, such as liposarcoma, where their amplifications are common drivers and form the basis for research into targeted therapies [43].

When these oncogene amplifications are carried on ecDNA, the result is increased intratumoural heterogeneity, as differences in ecDNA content between cells can lead to differences in oncogene expression [5,44].

### 4.2. Distribution of ecDNA Across Cancer Types

ecDNA has been detected across multiple cancer types. In a pan-cancer analysis, Kim et al. examined 3212 patients and found circular amplicons corresponding to ecDNA in 14.3% of tumours [19]. Bailey et al. conducted a study examining 14,778 patients with 39 different types of cancer. The study revealed that 17.1% of the tumour samples contained ecDNA, with its presence varying depending on the tissue/cancer type. ecDNA was particularly prevalent in cancers such as liposarcoma (54.9%), glioblastoma (49.1%), and HER2-positive breast cancer (46.4%). The authors also linked the frequency of ecDNA formation to environmental mutational processes, such as smoking [45].

#### The Occurrence of ecDNA in Lung Cancer

A study by Khandekar et al. examined 1216 cases of previously untreated lung cancer and found that ecDNA was present in 18.9% of cases. The incidence of ecDNA was similar in smokers and non-smokers. There were no significant differences in ecDNA prevalence in terms of gender, age, or most histological subtypes, except for carcinoids, where ecDNA was less common. Amplification of *MDM2* and other oncogenes in ecDNA was possibly responsible for lung cancer development in non-smoking patients. Moreover, patients harbouring ecDNA showed worse overall survival than other patients [46].

In their study, Pongor et al. presented a detailed analysis of ecDNA in SCLC. The authors demonstrated that ecDNA was responsible for high-level focal amplifications of key oncogenes in SCLC, as well as promoting the formation of gene fusions. By bringing oncogenes and transcription enhancers closer together, ecDNA enhances transcription in SCLC, potentially leading to a very high level of *MYC* expression. The study also highlighted the heterogeneous amplification of *MYC*, which was associated with the uneven segregation of ecDNA during cell division. Consequently, there was a rapid increase in tumour heterogeneity, as well as resistance to treatment and development of metastasis [40].

In another study, Choudhuri et al. highlighted the issue of SCLC initially being sensitive to chemotherapy but then acquiring resistance upon recurrence. The authors presented a case study in which resistance was acquired after treatment, and focal MYC amplification appeared on ecDNA simultaneously. ecDNA caused rapid quantitative variations in the *MYC* expression in the cell population through its action within a single tumour, forming cells with a greater or lesser number of *MYC* copies. This generates subpopulations of cells that are more resistant to DNA damage and, consequently, more capable of surviving subsequent lines of treatment [47].

In their work on SCLC, Behrouzi et al. emphasized the frequency of ecDNA in patients with this cancer. This contributed to tumour heterogeneity and resistance to chemotherapy. They also mentioned the possibility of detecting ecDNA in patients’ blood, which could be used for disease monitoring and patient stratification [48].

The study by Stensgaard et al. focused on non-small cell lung cancer (NSCLC). ecDNA was present in both healthy individuals and cancer patients. However, cancer patients had significantly higher levels of ecDNA. This study also highlights the usefulness of ecDNA as a potential diagnostic marker in NSCLC patients [49].

## 5. ecDNA and Cancer Progression

### 5.1. ecDNA-Driven Tumour Heterogeneity and Clonal Evolution

The mechanisms described above may contribute to tumour progression, clonal evolution and metastatic progression. Several investigations describe ecDNA as a major contributor to dynamic genetic heterogeneity, often exceeding the variability generated by classical chromosomal alterations [50,51].

Because ecDNA can rapidly change in copy number and chromatin configuration, subclones with a higher ecDNA copy number may gain growth and survival advantages, thereby supporting clonal selection and the emergence of more aggressive cellular phenotypes [52,53,54]. In addition, ecDNA molecules may undergo co-segregation during cell division, allowing multiple oncogenic ecDNA copies to be inherited together and accelerating tumour evolution.

Mechanistic studies further emphasize that ecDNA biogenesis and maintenance, including its formation through DNA damage-related processes and subsequent homeostasis, provide the molecular basis for its dynamic behaviour during cancer evolution [55].

### 5.2. ecDNA-Driven Therapy Resistance

The dynamic nature of ecDNA plays a significant role in tumour adaptation and drug resistance. Changes in ecDNA copy number enable rapid modulation of oncogene expression in response to therapeutic pressure. This genomic flexibility contributes to drug resistance and poor prognosis in many types of cancer, highlighting the importance of ecDNA as a powerful evolution mechanism in cancer progression [31].

This phenomenon has been demonstrated in glioblastoma, where ecDNA carrying oncogenes can dynamically increase or decrease in response to targeted therapies, leading to reversible drug resistance [37]. In patients treated with *EGFR* inhibitors, ecDNA containing mutant *EGFR* variants has been shown to disappear during treatment and re-emerge upon disease recurrence [56].

Similar mechanisms have been reported in other cancer types, including colorectal and lung cancer, where ecDNA-mediated oncogene amplification contributes to resistance to targeted therapies [40,57].

One of the most clinically important roles of ecDNA is its contribution to resistance against anticancer therapies, as demonstrated by experimental and clinical observations across multiple tumour types [58]. Reviews emphasize that ecDNA enables rapid adaptation to selective pressure imposed by chemotherapy and targeted therapies, partly through reversible changes in ecDNA copy number and transcriptional output [59,60].

This plasticity can enrich subclones carrying ecDNA that amplifies oncogenes or activate resistance-associated programmes, allowing these cells to survive treatment and eventually dominate the tumour population [17,58]. Recent studies also suggest that ecDNA may form BRD4-associated transcriptional hubs, in which the chromatin regulator BRD4 promotes strong enhancer-driven oncogene expression, further contributing to therapy resistance and tumour adaptation. Broader translational analyses also highlight emerging opportunities for ecDNA detection and therapeutic targeting in precision oncology [61].

Summing up, these results highlight that drug resistance induced by the presence of ecDNA is not limited to a single experimental system, but is consistently observed in cell culture models, animal models, and clinical samples, underscoring its significance for cancer therapy.

### 5.3. Role of ecDNA in Metastasis

Beyond local tumour evolution and therapy resistance, ecDNA may also contribute to metastatic progression. Growing evidence links ecDNA to aggressive disease features, including advanced tumour stage, poor clinical outcome, and enhanced genomic plasticity that supports tumour progression and dissemination [62].

ecDNA may increase invasiveness and metastatic potential by amplifying oncogenes involved in pathways regulating cell migration, angiogenesis, and survival in hostile microenvironments [63,64]. In addition, recent syntheses place ecDNA within a broader class of extrachromosomal circular DNA elements, emphasizing how open chromatin states and enhancer activity on ecDNA can drive strong transcriptional programmes associated with aggressive tumour behaviour and disease progression [65]. Furthermore, specific retention elements may facilitate ecDNA persistence within the nucleus, thereby stabilizing oncogenic ecDNA populations and supporting sustained tumour-promoting activity during cancer progression.

## 6. Use of Extrachromosomal DNA as a Prognostic Factor

### 6.1. ecDNA Amplification and Its Prognostic Implications in Patients with Cancer

Because ecDNA contributes to oncogene amplification, intratumoural heterogeneity and therapy adaptation, its presence may have prognostic significance in several tumour types [66,67]. For instance, the presence of ecDNA may soon become a reliable biomarker and diagnostic tool for various malignancies. However, it is crucial to understand that ecDNAs play diverse biological roles across different types of cancer, depending on the particular malignancy type. For instance, the prevalence of ecDNAs in lower-grade glioma, glioblastoma and subtypes of lung adenocarcinoma is high, hinting at a possible role of ecDNAs in carcinogenesis [34]. There are also notable differences between the distribution of ecDNA with sequences of different chromosomes. Sequences of chromosomes 1, 5, 7 and 8 represent higher content in ecDNAs in comparison to other chromosomes, which may explain the high amplification and enhancement of oncogenes *EGFR* and MYC in cancer tissues, e.g., which are bound mainly to the extrachromosomal amplicons [21]. As it was demonstrated across various studies, ecDNAs, especially in the form of mitochondrial DNA (mtDNA), are distributed unevenly between the cells, resulting in differentiated oncogenic potential and genomic diversity of offspring cells [68]. It is suggested that due to these properties, among others, ecDNAs can have an impact on prognosis and mortality of patients with various tumour types [36].

DeCarvalho et al. indicated a connection between intratumoral heterogeneity in glioblastoma and amplification of ecDNA in primary tumour samples, which resulted in compromising relapse-free time in patients [37]. In the case of small cell lung carcinoma, studies showed higher concentrations of ecDNAs composed of multiple genomic segments present in tumour cells in comparison to normal cells, where simple ecDNAs dominate. Prevailing genes found in ecDNAs in SCLC include *MYCL*, *MYCN*, and *KRAS*. The amplification of complex ecDNAs was attributed to worse overall survival (OS) in patients with SCLC. Moreover, the number of amplicons of ecDNAs was proven to vary significantly between primary sites of cancer and metastases, showing ecDNAs as a possible source of tumour heterogeneity and treatment resistance formation factor [69]. These findings are also evident in work by Choudhuri et al., where ecDNAs were detected in 18.8% of researched patient-derived xenografts [abbrev. PDX] models, with most of them having MYC-family gene amplification, with the most common of them being *MYCL* (33%), *MYCN* (22%) and *MYC* (17%). The data was obtained with the use of whole genome sequencing [abbrev. WGS] and processed with the AmpliconArchitect computational tool. To achieve clinical fidelity, profiles of SCLC PDX models were treated with three commonly used therapy routes (i.e., cisplatin with etoposide, topotecan and olaparib with temozolomide) and later compiled with patients’ previous treatment history. This way, the presence of cross-resistance in particular models was evaluated. Based on tumour biopsies, it was revealed that focal amplifications of *MYC*-family genes were present in the samples derived from premedicated patients with tumour relapse, yet absent in pretreatment samples. Based on comparative data, including PDX models, it was concluded that *MYC* paralogs located on ecDNAs are the main factor responsible for the presence of cross-resistance in relapsed tumour [47].

In breast cancer, the presence of ecDNA is generally considered a bad prognostic factor, contributing to the highly invasive nature of the tumour and development of treatment resistance. In the study conducted by Turner et al., patients with ecDNAs carrying oncogenes such as *EGFR* and *MYC* presented survival rates reduced up to 35% [13]. Amplification of the mentioned oncogenes is also believed to be responsible for more advanced stages of cancer at the moment of detection and elevated risk of metastases (up to 60%). Moreover, a larger number of ecDNA amplicons was found in highly invasive cell lines, e.g., triple-negative breast cancer, suggesting the role of ecDNA in the enhancement of tumour progression [63].

When it comes to prostate cancer, meta-analyses have shown increased expression of the *VAV2* oncogene within ecDNA. There was also a notable positive correlation between the concentration of ecDNA amplicons and the advancement of the tumour. Metastatic lymph nodes were also present more often in patients with high *VAV2* expression. Overall, studies have shown a correlation between high occurrence of *VAV2* within ecDNA and low survival rate of patients with prostate cancer, along with short disease-free survival (DFS) and progression-free survival (PFS) [64]. There are also significant findings that may, on the contrary, indicate positive prognostic qualities of genes coded by ecDNA. For instance, focal amplification of *ERBB2* and *CCNE1* has been associated with better prognosis in patients with gastric cardia adenocarcinoma who underwent surgical treatment in comparison to patients with lower amplification of these genes; however, the better survival tendency was reversed after 2 years of observation of the patients. The same studies show a strong correlation between high *EGFR* amplification and low survival rate in patients with gastric cardia adenocarcinoma. However, due to the relatively small research group of the study, there is a need for a re-evaluation of these findings in studies involving larger patient samples [65].

Overall, high concentration of ecDNAs in tumour cells promotes malignant progression of the cancer, as it allows transmission of the dominant traits to the offspring cells [67].

### 6.2. ecDNA-Related Drug Resistance Mechanisms

The properties of ecDNA, including a non-Mendelian inheritance pattern, serve as factors of intratumoral heterogeneity, promoting cancer cells’ adjustability to environmental variables and treatment [37]. ecDNAs contribute to drug resistance mainly by extrachromosomal expression and amplification of drug resistance-related genes. Depending on particular genes’ properties, drug resistance mechanisms include (1) modulation of ectopic expression of particular oncogenes (e.g., *EGFRvIII*) through alternative splicing; (2) regulation of functionality of genes related with drug targets; (3) dynamic vesicular ecDNA transfer between cancer cells and stroma; (4) reduction in intracellular drug accumulation by amplification of efflux transporters (e.g., ABCB1) [34].

#### 6.2.1. Modulation of Oncogenes’ Ectopic Expression

This property of ecDNA is mainly associated with the specific structure of the circular DNA, i.e., lack of higher order compaction except for the chromatinized domain core. As it promotes enhanced chromatin accessibility, genes encoded on ecDNA prove to be amplified in larger abundance in comparison to the same genes encoded on linear structures. As this fact allows higher chromatin interaction frequency at particular oncogenes’ loci, there is a bigger chance for modulation of expression of oncogenes on ecDNAs according to environmental changes. This phenomenon was elucidated in a study conducted on the properties of glioblastoma tumour cells. It was shown that these cells were capable of modulation of *EGFR* gene expression depending on patients’ *EGFR* tyrosine kinase inhibitor intake. This property is associated with ecDNA’s ability to modulate the number of EGFRvIII gene copies during metaphase, since the *EGFRvIII* gene is responsible for increased sensitivity to *EGFR* inhibitors. It is shown that *EGFR* mutations are provoked by the use of *EGFR*-targeted drugs, thus tumour cells gain significant growth capacity by selective removal of ecDNAs without the mutated *EGFR*, especially since the *EGFRvIII* gene seems to be present almost exclusively within ecDNA. Thus, the elimination of mutant *EGFR* would surpass the excessive growth of cancer cells [56].

#### 6.2.2. Gene Functionality Regulation

ecDNAs contribute to the development of chemotherapy resistance through the modification of the expression of drug target genes or the modification of their function. Mutations within genes carried by ecDNA, such as *KRAS*, *EGFR* and *MYCN*, contribute to resistance to apoptosis of tumour cells undergoing chemotherapy due to activation of signalling pathways, including MAPK and PI3K-AKT. A study on human ovarian cancer cell line UACC-1598 also showed selective loss of ecDNAs due to the use of gemcitabine, proving the major importance of the tumour’s extracellular instability as a survival factor and possible future target of research in terms of finding effective therapeutic solutions in oncological patients. Mentioned instability is concerned mainly with cells with a high abundance of ecDNAs, particularly with *MYCN* amplification [65,67,70]. On the contrary, treatment of SCLC tumour cells with methotrexate leads to a higher abundance of ecDNAs carrying dihydrofolate reductase gene (*DHFR*), which is responsible for methotrexate resistance. In this case, improved cell fitness is associated with an increase in the drug target. As the following cell line was placed in a drug-free medium, the *DHFR* expression in descendant cells decreased, resulting in cells showing increased methotrexate sensitivity [68].

#### 6.2.3. Dynamic Vesicular ecDNA Transfer

Intercellular heterogeneity aside, ecDNAs, especially mtDNAs, also take part in communication between neighbouring cells. As extracellular vesicles encapsulate ecDNA, it is possible to transport it between stroma and cancer cells. For instance, ecDNA transfer is evident as part of stroma-tumour crosstalk between cancer-associated fibroblasts (CAF) and breast cancer stem cells (CSC). It is shown that mtDNAs transferred to dormant tumour cells promote these cells’ activation, leading to resistance to endocrine therapy in breast cancer [68]. Similar mechanisms were discovered in pancreatic cancer models, as exchange of *TP53*-mutant mtDNAs between CAF and tumour cells proved to promote gemcitabine resistance [34].

#### 6.2.4. Amplification of Efflux Transporters

ecDNA can also be a carrier for drug resistance genes encoding drug efflux pumps. Such a situation occurs, e.g., in the case of chemoresistant neuroblastoma with high amplification of the *MYCN* oncogene along with upregulation of the *MRP* gene. The *MRP* gene encodes transmembrane glycoproteins responsible for the elimination of the drug from the affected tumour cells, making accumulation of the drug impossible [71].

As mentioned previously, excessive extrachromosomal DNA amplification within tumour cells contributes to intratumoral heterogeneity, increasing genetic diversity among offspring cells, thus promoting cells’ survival in an environment of high stress associated with medication. Alteration within ecDNA or thorough suppression of its replication may contribute to improvement in cancer cells’ drug sensitivity. However, further research regarding the impact of ecDNA on drug resistance in cancer cells is needed in order to provide more thorough insight into potential future therapeutic options targeting genes encoded in ecDNAs specifically. In case of stroma-tumour crosstalk via vesicular transfer, it is yet to be determined whether the mediation concerns mtDNA exclusively or other types of ecDNA are also involved. The particular mechanism of sorting and encapsulation of mtDNA within vesicles also requires further investigation.

## 7. Potential ecDNA-Targeted Therapeutic Strategies

The first way is to exploit transcription–replication conflict (TRC) and replication stress checkpoints. ecDNA shows widespread transcription with high TRC and replication stress, creating a selective dependence on S-phase checkpoint control. In preclinical models, genetic or pharmacologic CHK1 inhibition preferentially kills cells with ecDNA and can suppress the development of ecDNA-mediated resistance when combined with targeted therapy [72]. A first-in-human ecDNA-directed CHK1 inhibitor (BBI-355) is being evaluated clinically (ClinicalTrials.gov: NCT05827614) [73].

Mechanistically, highly amplified and actively transcribed ecDNA molecules increase the probability of collisions between replication and transcription machinery, leading to replication fork stalling and DNA damage. CHK1 is a central S-phase checkpoint kinase that helps stabilize stalled replication forks and prevents premature mitotic entry, which may explain why ecDNA-positive cells are particularly vulnerable to CHK1 inhibition [72,74].

The other way is to disrupt ecDNA transcriptional hubs and enhancer-driven overexpression. ecDNAs can accumulate into BRD4-tethered hubs that promote intermolecular enhancer–gene interactions. In these hubs, enhancers and oncogenes located on the same or different ecDNA molecules are brought into close spatial proximity, which amplifies transcriptional output beyond what would be expected from copy-number gain alone [36]. Because BRD4 contributes to the organization of these transcriptional hubs, BET inhibition (e.g., JQ1) may preferentially reduce ecDNA-derived oncogene transcription by disrupting enhancer–oncogene contacts rather than by acting only as a nonspecific transcriptional suppressor [36]. In pancreatic ductal adenocarcinoma, ecDNA with the MYC gene contributes to reversible dosage heterogeneity and adaptive plasticity, supporting the concept that interfering with ecDNA regulatory architecture may reduce rapid phenotypic transitions [75].

Another strategy is to target ecDNA maintenance in conjunction with DNA damage response (DDR) pathways. ecDNA replication can induce DNA double-strand breaks and activate ATM-mediated DDR, with evidence for reciprocal coupling between ecDNA maintenance and repair processes [76]. This coupling suggests that ecDNA-positive cells must continuously tolerate or repair DNA damage generated during ecDNA replication in order to preserve oncogene-containing circular DNA elements. Therefore, interference with selected DDR pathways could destabilize ecDNA maintenance or selectively increase replication-associated stress in ecDNA-positive tumour cells [65,76]. Complementarily, ecDNA biogenesis/repair studies highlight that distinct repair routes shape ecDNA formation and persistence, motivating exploration of synthetic-lethal combinations that selectively destabilize ecDNA while sparing chromosomal DNA repair capacity [65].

Interfering with ecDNA inheritance, co-segregation, and retention is also promising. In this context, “cooperative ecDNA species” refers to distinct ecDNA molecules within the same tumour cell that may carry complementary oncogenes or regulatory elements and are inherited together during mitosis [26]. This coordinated inheritance may preserve favourable combinations of ecDNA-driven traits, suggesting that effective therapeutic strategies may need to eliminate cooperating ecDNA populations rather than a single amplified oncogene circle [26]. Mechanistically, specific retention elements, including CpG-rich promoter regions, may tether ecDNA to mitotic chromosomes and thereby promote its transmission to daughter cells. The term “methylation-sensitive retention activity” refers to the observation that this tethering may depend on the epigenetic state of these elements, particularly DNA methylation; disrupting this process could promote ecDNA loss or mis-segregation over successive cell divisions [71].

Finally, blocking ecDNA-driven immune suppression could strengthen immunotherapy combinations. Large-scale analyses indicate that ecDNAs may amplify immunomodulatory genes and associate with reduced T-cell infiltration, linking ecDNA to an immune-suppressive tumour context [27]. Independently, ecDNA presence has been associated with signatures consistent with immune escape (including antigen presentation gene regulation), supporting exploration of ecDNA-depleting strategies as immunotherapy sensitizers [77].

## 8. Conclusions

Extrachromosomal DNA amplification is becoming a distinct and clinically relevant mechanism of tumour genome remodelling. Unlike conventional chromosome copy number increase, ecDNA enables exceptionally rapid and numerous focal amplifications of oncogenes.

Many studies discussed in this paper indicate that ecDNA is not a passive product of genomic instability, but an active factor in tumour evolution. Non-Mendelian inheritance and uneven segregation during mitosis contribute to increased cell heterogeneity within the tumour. Cell heterogeneity increases the tumour’s ability to adapt to environmental pressures, thereby promoting resistance to treatment, recurrence and metastatic potential. Consequently, ecDNA amplification is associated with a poorer prognosis in many types of cancer.

The detection of ecDNA is also a promising prognostic and predictive marker. However, its clinical implementation remains limited due to the lack of uniform detection methods. No single test offers scalability, structural resolution and wide availability at the same time. Before ecDNA can be introduced into widespread use, it will be necessary to develop widely available, reproducible tests for its detection.

The natural direction for further research should be the use of ecDNA in cancer therapy. Strategies exploiting ecDNA-associated replication stress and transcription–replication conflict, disrupting ecDNA transcription centres, disrupting maintenance/repair coupling, or promoting ecDNA loss may represent a breakthrough in recurrent and treatment-resistant cancers.

## Figures and Tables

**Table 1:** Summary of selected ecDNA detection methods.

Method	Principle	Advantages	Limitations
FISH	Direct visualization of amplified DNA signals in cells.	Confirms extrachromosomal localization; high specificity.	Low throughput; requires targeted probes [13]
WGS + AmpliconArchitect	Infers ecDNA from copy-number changes and breakpoint patterns.	Genome-wide; reconstructs complex amplicons.	Costly; computationally demanding; requiresvalidation [30,31]
Circle-seq	Enriches and sequences circular DNA molecules.	Sensitive screening for circular DNA.	Not specific for oncogenic ecDNA; requires confirmation [30,32]
CRISPR-CATCH	Targeted capture of selected ecDNA molecules.	High-resolution profiling of selected ecDNA.	Technically complex; limited routine availability [33]
RNA-seq–based prediction	Predicts ecDNA status from expression signatures.	Scalable; useful for screening large datasets.	Indirect; does not confirm ecDNA structure or localization [29]

## Data Availability

No new data were created or analyzed in this study. Data sharing is not applicable to this article.

## Abbreviations

The following abbreviations are used in this manuscript:

ATM	Ataxia-telangiectasia mutated
BET	Bromodomain and extraterminal domain
BFB	Break-fusion-bridge
BRD4	Bromodomain-containing protein 4
CCND1	Cyclin D1
CCNE1	Cyclin E1
CDK4	Cyclin-dependent kinase 4
CHK1	Checkpoint kinase 1
DDR	DNA damage response
DFS	Disease-free survival
DHFR	Dihydrofolate reductase
DM	Double minute
DMs	Double minutes
DNA	Deoxyribonucleic acid
eccDNA	Extrachromosomal circular DNA
ecDNA	Extrachromosomal DNA
EGFR	Epidermal growth factor receptor
EGFRvIII	Epidermal growth factor receptor variant III
ERBB2	Erb-B2 receptor tyrosine kinase 2
ERK	Extracellular signal-regulated kinase
FISH	Fluorescence in situ hybridization
GBM	Glioblastoma multiforme
HER2	Human epidermal growth factor receptor 2
HSRs	Homogeneously staining regions
JQ1	BET inhibitor JQ1
KRAS	Kirsten rat sarcoma viral oncogene homolog
MAPK	Mitogen-activated protein kinase
MDM2	Mouse double minute 2
MEK	MAPK/ERK kinase
mtDNA	Mitochondrial DNA
mTOR	Mechanistic target of rapamycin
MYC	MYC proto-oncogene
MYCL	MYCL proto-oncogene
MYCN	MYCN proto-oncogene
NGS	Next-generation sequencing
NSCLC	Non-small cell lung cancer
OS	Overall survival
PFS	Progression-free survival
PI3K	Phosphoinositide 3-kinase
RAF	Rapidly accelerated fibrosarcoma kinase
RAS	Rat sarcoma
RNA-seq	RNA sequencing
SCLC	Small cell lung cancer
SOX2	SRY-box transcription factor 2
TERT	Telomerase reverse transcriptase
TRC	Transcription–replication conflict
VAV2	Vav guanine nucleotide exchange factor 2
WGS	Whole-genome sequencing
